# Highly efficient homology-driven genome editing in human T cells by combining zinc-finger nuclease mRNA and AAV6 donor delivery

**DOI:** 10.1093/nar/gkv1121

**Published:** 2015-11-02

**Authors:** Jianbin Wang, Joshua J. DeClercq, Samuel B. Hayward, Patrick Wai-Lun Li, David A. Shivak, Philip D. Gregory, Gary Lee, Michael C. Holmes

**Affiliations:** Sangamo BioSciences, Inc., Richmond, CA 94804, USA

## Abstract

The adoptive transfer of engineered T cells for the treatment of cancer, autoimmunity, and infectious disease is a rapidly growing field that has shown great promise in recent clinical trials. Nuclease-driven genome editing provides a method in which to precisely target genetic changes to further enhance T cell function in vivo. We describe the development of a highly efficient method to genome edit both primary human CD8 and CD4 T cells by homology-directed repair at a pre-defined site of the genome. Two different homology donor templates were evaluated, representing both minor gene editing events (restriction site insertion) to mimic gene correction, or the more significant insertion of a larger gene cassette. By combining zinc finger nuclease mRNA delivery with AAV6 delivery of a homologous donor we could gene correct 41% of CCR5 or 55% of *PPP1R12C* (AAVS1) alleles in CD8^+^ T cells and gene targeting of a GFP transgene cassette in >40% of CD8^+^ and CD4^+^ T cells at both the *CCR5* and *AAVS1* safe harbor locus, potentially providing a robust genome editing tool for T cell-based immunotherapy.

## INTRODUCTION

Immunotherapy using gene-modified T cells for adoptive cell transfer is a rapidly expanding field that is currently being tested in early- and late-stage clinical studies, with recent successes seen in the treatment of hematologic malignancies using T-cell receptor or chimeric antigen receptor (CAR)-retargeted T cells (1–3). Engineered transgenes can be introduced into T cells to generate effector and memory CD8 and CD4 T lymphocytes specific for viral, fungal, bacterial, parasitic, and tumor-antigens and to design regulatory lymphocytes specific for the self-antigens responsible for autoimmune and inflammatory diseases (4). The rapid development of gene therapy in this field promises to enhance the function and safety of adoptive cellular therapies and opens the possibility for the development of novel, targeted therapies for the treatment of various diseases. *Ex vivo* T cell modification, with either a patient's cells or donor T cells, often utilizes integrating viral vectors such as lentivirus to confer long-lasting effects. However, the semi-random nature of vector insertion can result in non-authentic patterns of gene expression, transgene silencing over time, or the potential to trans-activate neighboring genes leading to insertional mutagenesis events (5–7). Current efforts in gene therapy are focused on safety improvements through changes in vector design or the use of genome editing technologies using targeted nucleases.

Targeted nucleases, which include zinc-finger nucleases (ZFNs), transcription activator-like effector nucleases (TALENs), and the RNA-guided clustered regularly interspaced short palindromic repeat (CRISPR)/Cas endonucleases, are a powerful class of enzymes that promote genome editing through the creation of a site-specific DNA double-strand break (DSB) at a pre-defined site in the genome (8). Repair of DSBs can proceed via the non-homologous end joining (NHEJ) or homology-directed repair (HDR) pathways (9,10). NHEJ is usually more efficient in mammalian cells and can be utilized mainly for gene disruption applications. HDR uses a homologous donor sequence as a template for the conservative repair of the DNA break and can be utilized for gene correction or gene addition, in addition to gene disruption. Given the markedly different genome editing outcomes, it would be desirable to exploit HDR for clinical applications to precisely correct a gene mutation *in situ*, or to specifically insert new genetic material at a pre-selected site including a safe harbor locus (11,12).

Gene disruption, which utilizes the error-prone NHEJ DNA repair pathway, can be achieved at relatively high levels in primary T cells (13–15). More recently, utilizing the HDR DNA repair pathway, small insertions became achievable by simultaneously delivering a targeted nuclease with a homology donor template as a single-strand oligonucleotide (16,17). However, such a powerful genome editing tool is limited to the introduction of small insertions or replacement of a short stretch of DNA sequence (<100 bp). This approach is impractical for inserting a transgene expression cassette into a predefined target locus. Previous studies utilizing plasmid or viral vectors such as IDLV to deliver the donor template demonstrated that targeted transgene addition can possibly be achieved in ∼5–7% of primary T cells (12,18). To improve the HDR efficiency and maximize the utility of HDR-based genome editing, we searched for more efficient ways to co-deliver the targeted nuclease and homology donor template.

In the present study, we evaluated the potential of adeno-associated virus (AAV) vectors to function as homology donors. By identifying AAV serotype 6 as a capsid variant with high tropism for human CD8^+^ and CD4^+^ T cells and combining this donor delivery method with mRNA expression of ZFNs, we were able to achieve unprecedented high levels of genome editing in both CD8 and CD4 T cells by homology-directed repair at pre-defined sites of the genome.

## MATERIALS AND METHODS

### ZFN reagents

ZFNs targeting the *CCR5* and *AAVS1* loci have been described previously (11,13). The following FokI variants were used to construct obligate heterodimeric versions of ZFNs (19,20): EL:KK (*CCR5*) and ELD:KKR (*AAVS1*). An optimized pair of the *AAVS1*-targeting ZFNs were used in this study (Supplementary Figure S1). The ZFN coding sequences were cloned into a modified version of plasmid pGEM4Z (Promega, Madison, WI, USA) containing a sequence of 64 alanines 3′ of the inserted gene sequence (21), which was linearized by SpeI digestion to generate templates for mRNA synthesis. mRNA was prepared using the mMESSAGE mMACHINE^®^ T7 ULTRA Kit (Life Technologies, Carlsbad, CA, USA) or by TriLink Biotechnologies (San Diego, CA, USA).

### AAV vectors

All AAV vectors were produced at Sangamo BioSciences as described below. *CCR5* and *AAVS1* homologous donor templates (12,22) were cloned into a customized plasmid pRS165 derived from pAAV-MCS (Agilent Technologies, Santa Clara, CA, USA), containing AAV2 inverted terminal repeats (ITRs), to enable packaging as AAV vectors using the triple-transfection method (23). Briefly, HEK 293 cells were plated in 10-layer CellSTACK chambers (Corning, Acton, MA, USA), grown for 3 days to a density of 80%, then transfected using the calcium phosphate method with an AAV helper plasmid expressing AAV2 Rep and serotype specific Cap genes, an adenovirus helper plasmid, and an ITR-containing donor vector plasmid. After 3 days the cells were lysed by three rounds of freeze/thaw, and cell debris removed by centrifugation. AAV vectors were precipitated from the lysates using polyethylene glycol, and purified by ultracentrifugation overnight on a cesium chloride gradient. Vectors were formulated by dialysis and filter sterilized.

### Gene editing of T cells

Fresh purified CD8^+^ and CD4^+^ T cells were purchased from AllCells.com and maintained in X-VIVO 15 (Lonza, Basel, Switzerland) supplemented with 10% FBS, 2 mM l-glutamine, 1% penicillin/streptomycin/amphotericin B (PSA) (Sigma Aldrich, St Louis, MO, USA), 20 ng/ml IL2 (PeproTech, Rocky Hill, NJ, USA), and anti-CD3/CD28-beads (Life Technologies, Grand Island, NY, USA). T cells were cultured for a full day and then transduced with AAV vectors at the indicated vector genome (vg) copy per cell in maintenance media for 16 h. The cells were washed 2–3 times with PBS then resuspended in BTXpress high performance electroporation solution (Harvard Apparatus, Holliston, MA, USA) to a final density of 2–4 × 10^6^ cells/ml. This cell suspension was mixed with 40 (*AAVS1*, each individual ZFN) or 60 μg/ml (*CCR5*, 2A linked ZFN) or other amount as indicated of *in vitro* transcribed ZFN mRNA and electroporated in a BTX ECM830 Square Wave electroporator (Harvard Apparatus) in a 2 mm cuvette using a single pulse of 250 V for 5 ms. Electroporated cells were maintained in the X-VIVO 15 culture medium with the addition of 20 ng/ml IL7 (PeproTech, Rocky Hill, NJ, USA).

### Analysis of gene modification

For experiments using GFP donors, cells were collected at different time points post-treatment and analyzed for GFP expression using a Guava EasyCyte 5HT (EMD Millipore, Billerica, MA, USA). Data acquired was analyzed using InCyte version 2.5 (EMD Millipore). In addition, a semi-quantitative PCR was used to measure GFP integration at the *CCR5* locus. Briefly, a primer present in the polyA region of the GFP cassette and a primer located 3′ (outside) of the end of the right *CCR5* homology arm region such that one primer bound only within the donor and the other bound to genomic DNA outside of the donor, were used to generate a PCR product (Supplementary Table S1, *CCR5* in-out primers). This was compared to the product resulting from a control primer set recognizing sequences in the *CCR5* locus located 5′ to the left homology donor. The control primer set (Supplementary Table S1, *CCR5* control primers) only binds genomic DNA and was therefore used to normalize DNA input. The concentration of the In-Out primer was 2 times that of the control primer set, to increase detection sensitivity. The relative intensities of each band were compared to a previously quantitated standard set generated from a pool of gDNA isolated from a K562 cell line with a constant level of GFP integration at the *CCR5* locus as quantitated by Southern blot. To detect GFP integration at the *AAVS1* locus, PCR products amplified with the *AAVS1* out-out1 primers (Supplementary Table S1) were separated on 1% agarose gel. Genomic DNA isolated from a K562 clone with GFP integration at the *AAVS1* locus and its dilutions were also amplified side-by-side for comparison. However, the PCR is not quantitative since amplification of the large GFP-TI PCR products is very inefficient compared to the small wild-type product (Supplementary Figures S5 and S7C).

For experiments using RFLP donors, restriction fragment length polymorphism (RFLP) assays and Illumina deep sequencing were used to quantify the frequency of genome modification. The RFLP assay has been previously described (22). Briefly, the target site was PCR amplified with primers located outside of the homologous arms as listed in Supplementary Table S1 (out-out1 primers), digested with XhoI (CCR5 RFLP) or HindIII (AAVS1 RFLP) restriction enzyme, and then separated in gel by electrophoresis. To obtain a relatively more accurate estimation of the overall RFLP frequency in case the digestion was incomplete, the undigested band was gel purified and processed for deep sequencing as described below to quantify RFLP frequency of the undigested band. The result was used to calculate adjusted RFLP frequency. For Illumina deep sequencing, gel-purified Out-Out1 PCR products (undigested band or untreated) were amplified with a target-specific Miseq adaptor primer pair (Supplementary Table S1, adaptor primers) and sequence barcodes were added in the subsequent PCR reaction using the barcode primer pairs. Alternatively, 1/5000 of the PCR products amplified using primer pair out-out1 were re-amplified with primer pair out-out2 (Supplementary Table S1), then 1/5000 of the second PCR products were amplified using the Miseq adaptor primers. The final PCR products were cleaned and sequenced in an Illumina Miseq sequencer, essentially as described by the manufacturer (Illumina, San Diego, CA, USA). For analysis of gene modification levels, a custom-written computer script was used to merge paired-end 150 bp sequences, and adapter trimmed via SeqPrep (John St. John, https://github.com/jstjohn/SeqPrep, unpublished). Reads were aligned to the wild-type template sequence. Merged reads were filtered using the following criteria: the 5′ and 3′ ends (23 bp) must match the expected amplicon exactly, the read must not map to a different locus in the target genome as determined by Bowtie2 (24) with default settings, and deletions must be <70% of the amplicon size or <70 bp long. Indel events in aligned sequences were defined as described previously (25), with the exceptions that indels of 1 bp in length were also considered true indels to avoid undercounting real events, and true indels must include deletions occurring within the sequence spanning between the penultimate bases (adjacent to the gap) of the binding site for each partner ZFN. Events with expected RFLP modification were defined based on perfect alignment with the DNA sequence containing the novel restriction site (*CCR5* RFLP: AGTTTGTCTCGAGGTGATGA; *AAVS1* RFLP: AGTGGGGCAAGCTTTACTAGGG) of the expected sequences.

### Statistical analysis

All statistical analyses were performed using the Excel Analysis ToolPak.

## RESULTS

**Figure 1.**
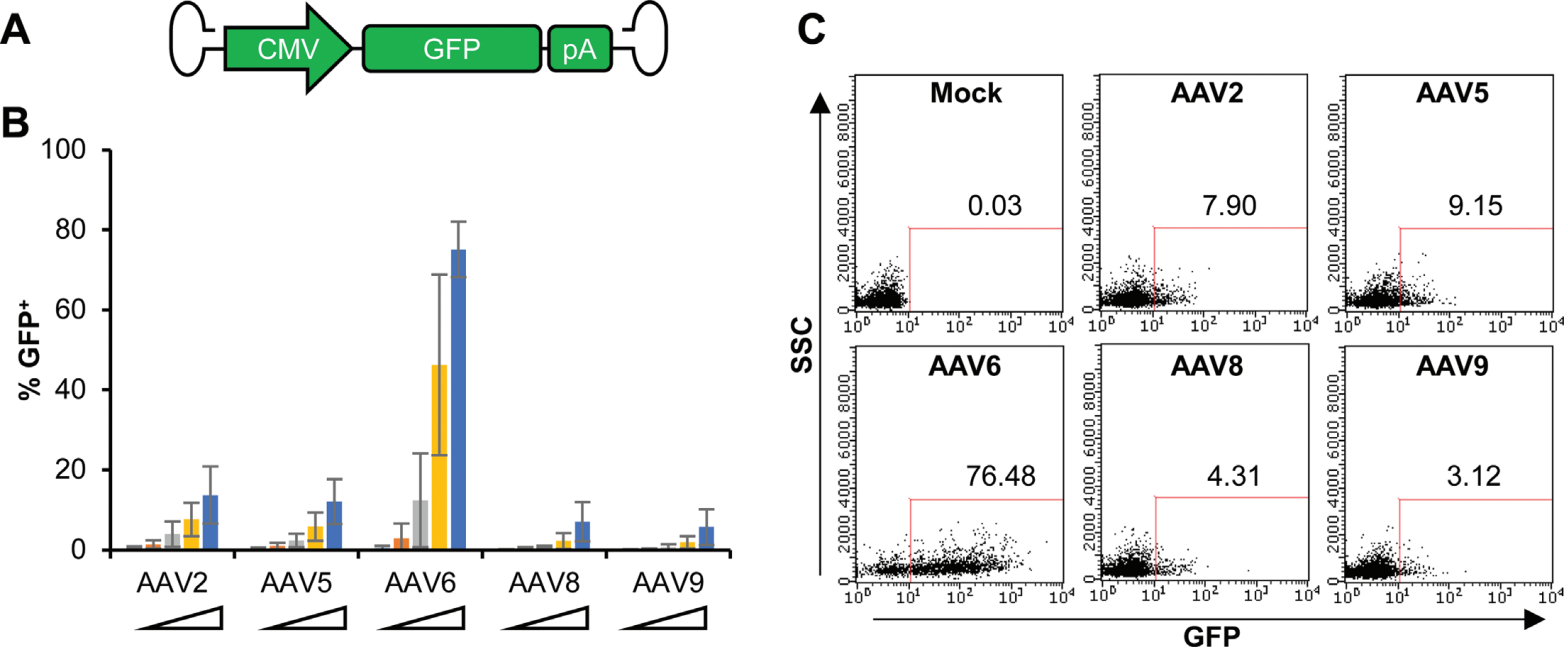
CD8^+^ T cells are efficiently transduced by AAV serotype 6. (**A**) Schematic of recombinant AAV vector genomes containing CMV promoter-driven eGFP expression cassette. Inverted terminal repeats (ITRs) are derived from AAV2. Different serotype specific Cap genes were used to generate the corresponding recombinant AAV vectors as described in Materials And Methods. (**B**) CD8^+^ T cells were transduced with increasing doses of GFP-expressing AAV vectors of the indicated serotypes, and GFP expression was determined at 5 days post-transduction by flow cytometry. The vector doses used were 1 × 10^4^, 3 × 10^4^, 1 × 10^5^, 3 × 10^5^, 1 × 10^6^ vector genomes (vg)/cell. Combined results of 3 independent experiments using different CD8^+^ T cell donors were shown as mean ± SD. (**C**) Flow cytometric plots from a representative experiment at 1 × 10^6^ vg/cell are shown.

To evaluate the use of AAV vectors to promote homology-driven genome editing in T cells, we first compared the ability of different AAV capsid serotypes to transduce these cells. To evaluate the gene transfer efficiency of these different AAV serotypes in T cells, we used vectors encoding a GFP expression cassette (Figure 1A) to transduce both CD8^+^ (Figure 1B and C) and CD4^+^ (Supplementary Figure S2) T cells. For both T cell subsets, we found that AAV serotype 6 gave the highest rates of transduction across a range of vector doses, reaching >75% transduced cells at the higher doses used, suggesting T cells are highly permissive to AAV6 transduction.

**Figure 2.**
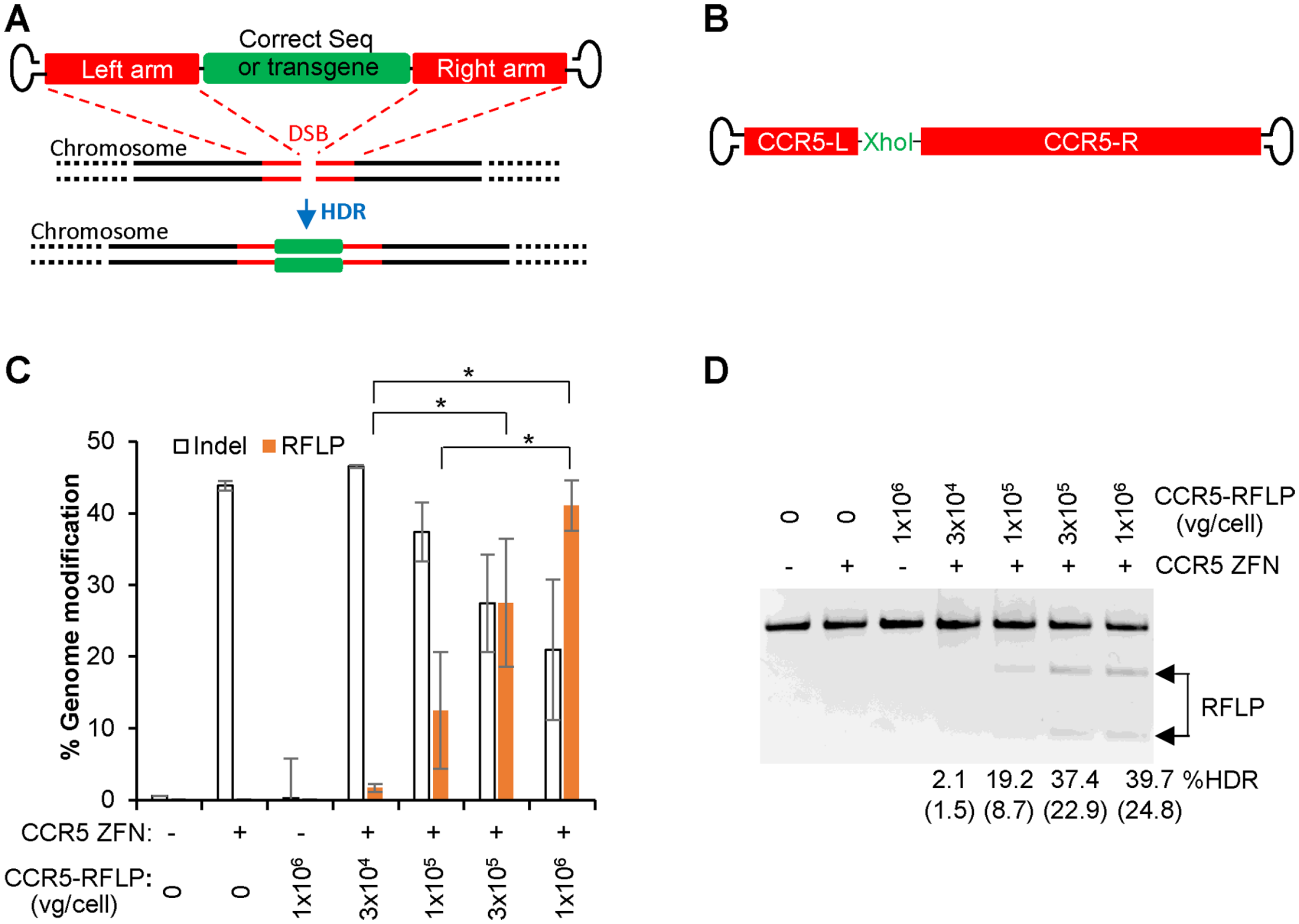
Combination of ZFN mRNA and AAV6 vectors promotes high levels of gene correction-like events at the *CCR5* locus in CD8^+^ T cells. (**A**) Schematic showing use of AAV vector as a template for homology directed repair (HDR) of a double-strand break (DSB), as induced by target-specific nucleases, to introduce a corrected gene sequence or transgene into a predetermined genomic target site. (**B**) Schematic of AAV vector genomes containing *CCR5*-RFLP homology donors. L (left) and R (right) refer to *CCR5* genomic sequences, comprising 509 and 1349 bp respectively. (**C**) CD8^+^ T cells were transduced with AAV6 vectors carrying the *CCR5*-RFLP donor at indicated doses for 16 hours, then electroporated with *CCR5* ZFN mRNA (60μg/ml). Cells were analyzed 7 days post-electroporation by deep sequencing to measure the efficiency of genome modification (% indels and RFLP). Combined results of three experiments using three different CD8^+^ T cell donors are shown. Mean ± SD. *P < 0.05, two-tailed *t*-test to compare %RFLP between conditions with different doses of AAV6 donor in the presence of ZFN mRNA treatment. (**D**) Dose-dependent insertion of XhoI site at *CCR5*, confirmed by RFLP analysis. Adjusted%RFLP HDR are shown below the lanes with visible RFLP bands based on deep sequencing results of the undigested top band due to incomplete digestion. The %RFLP before adjustment are shown in parentheses. One representative experiment is shown.

**Figure 3.**
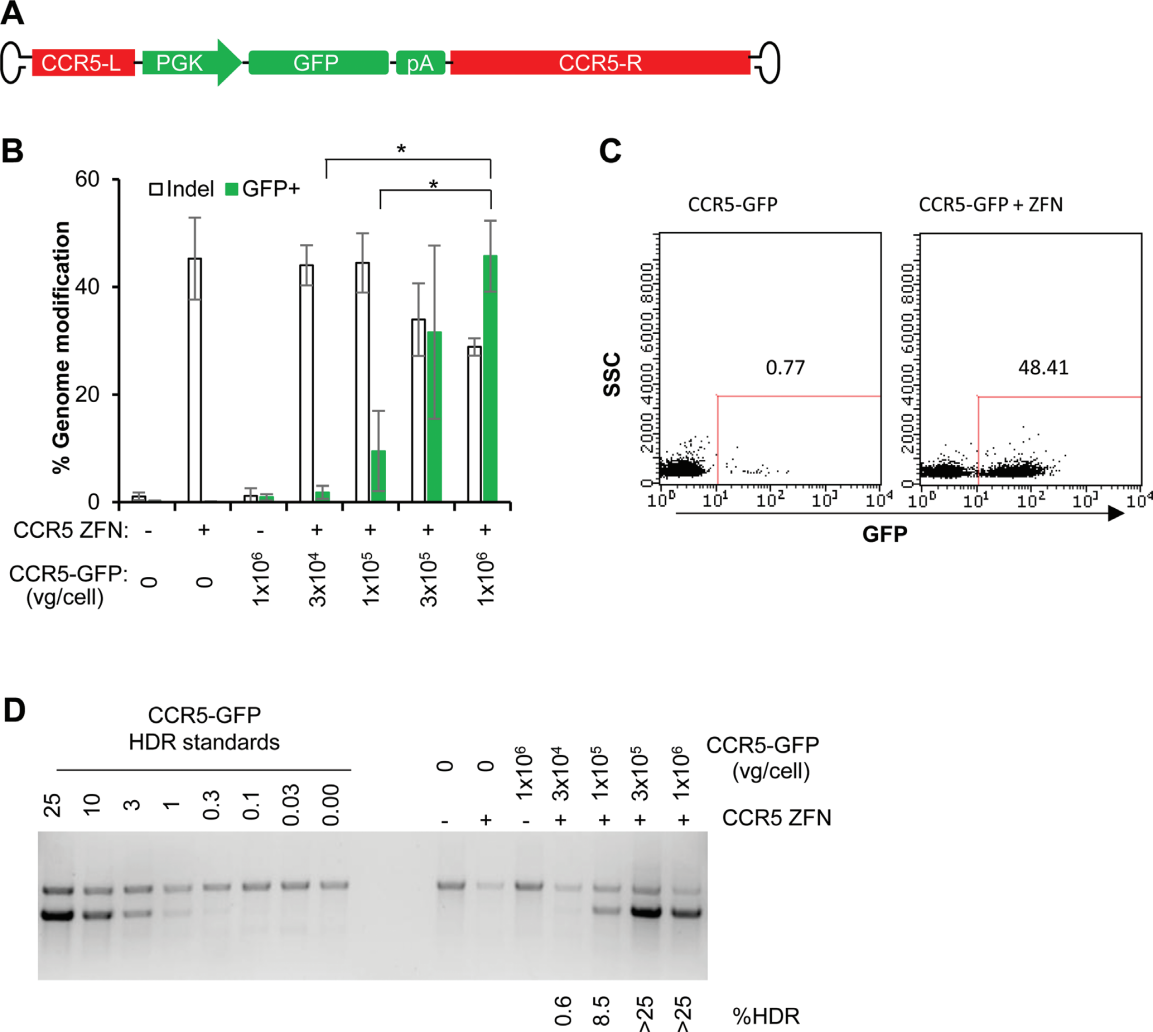
Combination of ZFN mRNA and AAV6 vectors promotes high levels of transgene addition at the *CCR5* locus in CD8^+^ T cells. (**A**) Schematic of AAV vector genomes containing *CCR5*-GFP homology donors. L (left) and R (right) refer to *CCR5* genomic sequences, comprising 476 and 1428 bp, respectively. (**B**) CD8^+^ T cells were treated as described in Figure 2C, but using *CCR5*-GFP donor vectors, with and without *CCR5* ZFN mRNA electroporation. Cells were collected at 14 days post-transduction and analyzed by flow cytometry for %GFP^+^, and by deep sequencing to measure% indels. Results were combined from three experiments using three different CD8+ T cell donors. Mean ± SD. *P < 0.05, two-tailed *t*-test to compare%GFP^+^ between conditions with different doses of AAV6 donor in the presence of ZFN mRNA treatment. (**C**) Flow cytometry plots from one representative experiment were shown using 1 × 10^6^ vg/cell *CCR5*-GFP donor, at 14 days post-electroporation. (**D**) Confirmation of targeted integration of GFP expression cassette at the *CCR5* locus by semi-quantitative PCR. The %GFP HDR was estimated by comparison to standards.

We then examined the ability of AAV6 vectors to promote HDR-mediated genome editing in T cells. A double-strand break (DSB) can be introduced into a predetermined genomic site by target-specific nucleases, which can be repaired by the cellular HDR machinery in the presence of a homology template/donor possibly delivered as AAV vector, to introduce a corrected gene sequence or transgene into the intended target site (Figure 2A). To stimulate HDR in CD8^+^ T cells, sixteen hours after transduction of the cells with AAV6 donor vectors, a site-specific double-stranded DNA break (DSB) was introduced at the chemokine (C–C motif) receptor 5 (*CCR5*) locus by delivering a previously characterized *CCR5* ZFN pair (13) as mRNA via electroporation. Two different homology donor templates were evaluated, representing both minor gene editing events (restriction site insertion) to mimic gene correction, or the more significant insertion of a larger gene cassette. In each case, the AAV6 vectors contained the same homologous *CCR5* sequences, flanking, either an XhoI restriction site (*CCR5*-RFLP, Figure 2B) or a GFP expression cassette (*CCR5*-GFP, Figure 3A). Successful gene editing events were determined by population deep sequencing and RFLP analysis to detect XhoI insertion, or by flow cytometry and semi-quantitative PCR for site-specific GFP addition.

NHEJ- and HDR-mediated repair are competitive events, and we found that increasing the dose of the *CCR5*-RFLP donor vector in the presence of *CCR5* ZFNs led to an increase in the rate of XhoI insertion at the *CCR5* locus, resulting in 41% of alleles being modified by the HDR pathway as detected by Illumina deep sequencing (Figure 2C and Supplementary Table S2). This was accompanied by a corresponding decrease in the extent of the characteristic NHEJ-mediated indels. The high levels of gene correction-like events were also confirmed by RFLP analysis (Figure 2D), in which the target site was PCR amplified with primers located outside of the homologous arms, digested with XhoI restriction enzyme, and then separated in gel by electrophoresis. To avoid problems caused by incomplete digestion with restriction enzyme, which can potentially occur due to the annealing of a RFLP-containing sequence to a wild-type or indel sequence during PCR reactions, we gel purified the top band and processed it for Illumina deep sequencing. The results were used to calculate the adjusted %RFLP HDR (Figure 2D), which is comparable with the results shown in Figure 2C, confirming that combination of ZFN mRNA and AAV6 donor can promote high levels of site-specific gene correction-like events at the *CCR5* locus in CD8^+^ T cells.

Similarly in CD4^+^ T cells, we also achieved greater than 30% of alleles being modified by the HDR pathway using 1 × 10^6^ vg/cell of the *CCR5*-RFLP donor vector in the presence of *CCR5* ZFNs as detected by Illumina deep sequencing (Supplementary Figure S3A) and RFLP analysis (Supplementary Figure S3B).

To evaluate whether a full transgene expression cassette, comprised of a promoter sequence, an open reading frame, and a polyadenylation site, can be efficiently integrated at a pre-defined genome site by HDR, we performed similar experiments using a *CCR5*-GFP donor vector (Figure 3A). Increasing levels of stable GFP expression were observed following transduction by increasing doses of the *CCR5*-GFP donor vector, but only when the ZFN mRNA was also delivered (Figure 3). This resulted in >45% of the CD8^+^ cells being GFP^+^ when AAV6 donor was used at 1 × 10^6^ vg/cell (Figure 3B and C). The high levels of targeted transgene addition by HDR was also confirmed by a semi-quantitative PCR (Figure 3D), suggesting that combination of ZFN mRNA and AAV6 donor can promote high levels of site-specific transgene addition at the *CCR5* locus in CD8^+^ T cells.

Similar to CD8^+^ T cells, >40% GFP^+^ cells were observed in CD4^+^ T cells treated with the combination of *CCR5*-GFP donor and ZFN mRNA delivery (Supplementary Figure S4A, B), which was also confirmed by a semi-quantitative PCR (Supplementary Figure S4C).

**Figure 4.**
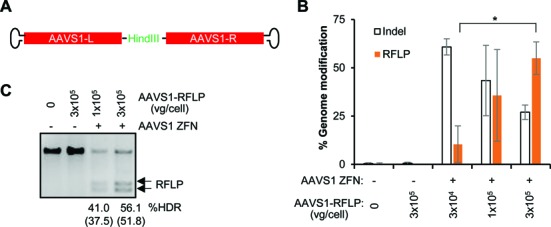
AAV6 vectors and ZFN mRNA promote high levels of gene correction-like events at the *AAVS1* locus in CD8^+^ T cells. (**A**) Schematics of AAV6 vectors used to deliver *AAVS1* homology donors. L (left) and R (right) refer to *AAVS1* genomic sequences, comprising 789 and 837 bp respectively (11). (**B**) CD8^+^ T cells were transduced with AAV6 vectors carrying the *AAVS1*-RFLP donor at indicated doses (vg/cell) for 16 h and/or electroporated with *AAVS1* ZFN mRNA. Cells were analyzed 7 days post-electroporation by Illumina deep sequencing to measure the efficiency of genome modification (% indels and RFLP). Results are combined from three experiments using three different CD8^+^ T cell donors. Mean ± SD. *P < 0.05, two-tailed *t*-test to compare%RFLP between conditions with different doses of AAV6 donor in the presence of ZFN mRNA treatment. (**C**) Confirmation of insertion of HindIII site at the *AAVS1* locus by RFLP assay. Adjusted%RFLP HDR are shown below the lanes with visible RFLP bands based on deep sequencing results of the undigested top band due to incomplete digestion. The %RFLP before adjustment are shown in parentheses. One representative experiment is shown.

**Figure 5.**
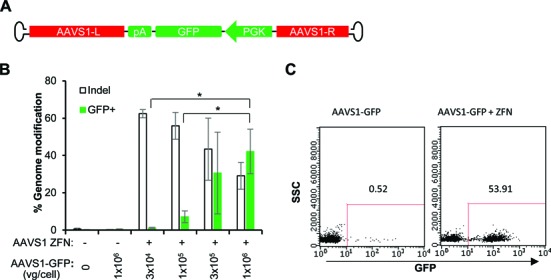
AAV6 vectors and ZFN mRNA promote high levels of transgene addition at the *AAVS1* locus in CD8^+^ T cells. (**A**) Schematics of AAV6 vectors used to deliver *AAVS1*-GFP homology donors. L (left) and R (right) refer to *AAVS1* genomic sequences, comprising 789 and 570 bp, respectively. (**B**) CD8^+^ T cells were treated as described in Figure 4B, but using *AAVS1*-GFP donor. Cells were collected 14 days post-transduction and analyzed by flow cytometry for %GFP^+^, and by deep sequencing to measure% indels. Results were combined from three experiments using three different CD8^+^ T cell donors. Mean ± SD. *P < 0.05, two-tailed *t*-test to compare %GFP^+^ between conditions with different doses of AAV6 donor in the presence of ZFN mRNA treatment. (**C**) Flow cytometry plots from a representative experiment using 1 × 10^6^ vg/cell *AAVS1*-GFP donor at 14 days post-electroporation.

To establish the generality of these results we performed an analogous set of studies using reagents specific for the protein phosphatase 1, regulatory subunit 12C (*PPP1R12C*) gene, a ‘safe harbor’ locus known as *AAVS1* (11,12). In CD8^+^ T cells, insertion of a HindIII restriction site occurred, on average, at 55% of the *AAVS1* alleles (Figure 4 and Supplementary Table S3), while GFP transgene addition was observed at an average rate of >42% of the cells (Figure 5, Supplementary Figure S5). Similar high rates of gene editing at *AAVS1* were achieved in CD4^+^ T cells, with insertion of the HindIII restriction site observed at >45% of the *AAVS1* alleles (Supplementary Figure S6) and stable GFP addition detected in >45% of the cells (Supplementary Figure S7). Taken together these results demonstrate that AAV6 vectors are an effective vehicle for delivering donor DNA templates to T cells, and that when combined with introduction of ZFN via mRNA electroporation, both minor gene editing events (mimic of gene correction) and larger gene additions are observed at high efficiency.

## DISCUSSION

We have identified AAV serotype 6 as a capsid variant with high tropism for both human CD8^+^ and CD4^+^ T cells and demonstrated that combination of ZFN mRNA delivery with AAV6 delivery of a homologous donor can result in high levels of both minor gene editing events (mimic of gene correction) and larger gene additions. Such a platform for donor delivery acts downstream of DSB formation and it is likely that the vectors will have similar utility as partners for all classes of targeted nucleases.

CD8^+^ cytotoxic T lymphocytes (CTLs) have been proven to exhibit potent anti-tumor activity in preclinical and clinical studies (1–2,26–28). The technology described here can be utilized for highly efficient editing of CTLs to potentially enhance their activity and safety via the insertion of genes at a specific pre-selected site such as a safe harbor locus (11,12). In addition, the ability to simultaneously disrupt and insert genes can also facilitate the development of combined therapeutic approaches. The above mentioned transgene addition can be combined with simultaneous gene disruption. For example, disruption of genes in T cells that play a role in suppression of effector activity, such as programmed cell death 1 (*PDCD1*, also known as *PD1*), cytotoxic T-lymphocyte-associated protein 4 (*CTLA4*), and others, to make genetic engineered T cells less susceptible to the immune evasion mechanisms of tumor cells or to the inhibitory tumor environment, to improve anti-tumor activity (4,29–35), disruption of endogenous TCR to enhance specificity of re-targeted T cells (36), disruption of TCR or MHC to generate universal donor cells (37,38).

CD4^+^ T cells play a pivotal role in immunosurveillance. In addition to classic helper function, CD4^+^ T cells also have cytolytic activity depending on class II-restricted recognition of tumor antigens, and have exhibited anti-tumor effects in patients with metastatic melanoma (39–43). Furthermore, Engineered CD19-specific CD4^+^ T cell responses comprised at least part of the anti-tumor effects against B-cell malignance in a few of the recent successful clinical trials (26-28). Besides this promising anti-tumor function, genome edited CD4^+^ T cells have been tested as an adoptive cell therapy for HIV/AIDS (44). Hence, the use of this technology for highly efficient genome editing will further enhance the development of T cell immunotherapies.

Targeted nucleases provide a mechanism to achieve more precise genome editing, which may be especially important when the cellular target is a long-lived cell such as a hematopoietic stem cell or memory T cell. In addition to engaging the homology-directed repair pathways to achieve precise on-target genome editing, AAV genomes can also become inserted at the site of a DSB through NHEJ-mediated end capture events (45–47). Such events can be considered on-target gene addition when occurring at the intended nuclease target site, but are off-target events when occurring at DSBs generated by either random cellular events, or at the sites of off-target nuclease activity. To investigate which mechanism was responsible for the high levels of stable GFP expression observed in T cells following the introduction of both ZFNs and AAV6 donor, we combined ZFN mRNA treatment with matched or mismatched AAV6 vectors encoding a GFP expression cassette and monitored GFP expression 9 days later (Supplementary Figure S8). The results confirmed that HDR is the predominant DNA repair pathway used for genome editing T cells when combining ZFN mRNA and AAV6 donor delivery. In addition, a majority of the non-HDR events are most likely end-capture of the AAV donor at the on-target site. Therefore, the approach described here provides a marked improvement over other non-nuclease/non-targeted integration approaches, such as those utilizing AAV vector alone or lentiviral vectors, which effectively result in 100% off-target integration.

To achieve HDR-mediated gene editing at levels that are likely to be of therapeutic benefit requires optimization of several steps. First, a well-tolerated process must be developed to introduce a targeted nuclease into the cell to create a DSB at sufficiently high levels. Delivery of ZFN as mRNA by electroporation appears to be very efficient with minimal cytotoxicity (14,16). In addition, a homologous donor template must also be introduced. An optimal donor construct should be selected by designing and testing several donor variants, including varying homology arm lengths, to ensure the chosen construct provides efficient HDR-driven genome editing. The size and position of the homology arms is especially important if the intended target site for gene correction or transgene addition is distant from the nuclease cleavage site, as this can lead to substantially reduced levels of HDR-mediated genome editing (48–50). This donor template can be single- or double-stranded DNA, or short oligonucleotides (16,17). Given AAV-based vectors can efficiently package genomes up to ∼4.7 kb (51), this makes AAV vector well suited for both gene correction and transgene addition, whereas due to size limitations single strand oligonucleotides are best suited for gene correction or insertion of small DNA fragments as described previously (16,17). The use of plasmid DNA as donor in T cells can be poorly tolerated. In contrast, the combination of AAV6 donor and mRNA ZFN approach is well tolerated and genome modified cells grow indistinctively as unmodified cells as evidenced by stable levels of %RFLP HDR or %GFP^+^ cells after more than two weeks *in vitro* expansion (Supplementary Figure S9). AAV6 vector as donor is also superior to plasmid DNA and integrase-deficient lentivirus (IDLV), which supported much lower levels of HDR-mediated genome editing (Supplementary Figures S10 and S11). It is possible that single-stranded DNA is more recombinogenic and the higher levels of HDR-driven genome editing observed in the current study is due to the single-stranded genome of AAV. However, comparable peak levels of HDR between cells treated with either single-strand or self-complementary (double-stranded) AAV (51) donor vector (Supplementary Figure S12) do not support such a hypothesis. In addition, we found that transducing the cells with AAV6 donor under serum-free conditions (4 hours) resulted in a marked improvement in HDR-mediated genome editing at significantly lower vector doses (2-log lower doses of AAV6 donor), making the large-scale, clinical production of genome edited T cells more feasible (Supplementary Figure S13). Finally the template must engage the HDR machinery, which is most active during the S and G2 phases of the cell cycle. In this regard, AAV preferentially transduce cells in S phase (52). Furthermore, T lymphocytes represent the ideal cell type for genome editing via HDR, since T cells can be easily obtained through apheresis, purified, and then robustly expanded *ex vivo*, allowing HDR to occur, prior to adoptive cell transfer.

In summary, our data suggest that T cell-tropic AAV vectors can support unprecedented high levels of HDR-mediated genome editing including both gene correction (small nucleotide changes) and targeted gene addition strategies in both primary CD8^+^ and CD4^+^ T cells, which could have broad applications in the development of improved T cell immunotherapies for a broad range of indications, including cancer, chronic infection and autoimmunity.

## ACCESSION NUMBERS

Illumina deep sequencing data have been deposited in the NCBI Sequence Read Archive (SRA, http://www.ncbi.nlm.nih.gov/Traces/sra) with accession number SRP060624.

## Supplementary Material

SUPPLEMENTARY DATA
