# Supplementary material for: Highly efficient homology-driven genome editing in human T cells by combining zinc-finger nuclease mRNA and AAV6 donor delivery
Source: Nucleic Acids Res. 2015 Nov 2;44(3):e30. doi: 10.1093/nar/gkv1121 (PMC4756813; doi:10.1093/nar/gkv1121)
Supplement: SUPPLEMENTARY DATA [file supp_44_3_e30__index.html]

Highly efficient homology-driven genome editing in human T cells by combining zinc-finger nuclease mRNA and AAV6 donor delivery — SUPPLEMENTARY DATA 

# Highly efficient homology-driven genome editing in human T cells by combining zinc-finger nuclease mRNA and AAV6 donor delivery

## SUPPLEMENTARY DATA

- SUPPLEMENTARY DATA
