# Supplementary material for: Highly efficient homology-driven genome editing in human T cells by combining zinc-finger nuclease mRNA and AAV6 donor delivery
Source: Nucleic Acids Res. 2015 Nov 2;44(3):e30. doi: 10.1093/nar/gkv1121 (PMC4756813; doi:10.1093/nar/gkv1121)
Supplement: SUPPLEMENTARY DATA [file supp_gkv1121_Supplementary_Materials_jw102315_updated.pdf]

***Supporting Supplementary Material for:***

**Highly Efficient Homology-driven Genome Editing in Human T Cells by Combining Zinc-finger Nuclease mRNA and AAV6 Donor Delivery**

Jianbin Wang[1], Joshua J. DeClercq, Samuel B. Hayward, Patrick Wai-Lun Li, David A. Shivak, Philip D. Gregory, Gary Lee, and Michael C. Holmes

Sangamo BioSciences, Inc., Richmond, CA, USA.

[1]Correspondence should be directed to:

Jianbin Wang, PhD
Sangamo Biosciences, Inc.
Richmond, CA 94804, USA.
Phone: (510) 970 7829
email: jwang@sangamo.com

**This file contains**: Supplementary Figures S1-12 and Supplementary Table S1-3.

# Supplementary Figures

### Supplementary Figure S1

**AAVS1 ZFN SBS#30035 (NELD):**

```
  1 MDYKDHDGDY KDHDIDYKDD DDKMAPKKKR KVGIHGVPAA MAERPFQCRI CMRNFSRSDH
 61 LSRHIRTHTG EKPFACDICG RKFATSGHLS RHTKIHTGSQ KPFQCRICMR NFSYNWHLQR
121 HIRTHTGEKP FACDICGRKF ARSDHLTTHT KIHTGSQKPF QCRICMRNFS HNYARDCHIR
181 THTGEKPFAC DICGRKFAQN STRIGHTKIH LRGSQLVKSE LEEKKSELRH KLKYVPHEYI
241 ELIEIARNST QDRILEMKVM EFFMKVYGYR GKHLGGSRKP DGAIYTVGSP IDYGVIVDTK
301 AYSGGYNLPI GQADEMERYV EENQTRDKHL NPNEWWKVYP SSVTEFKFLF VSGHFKGNYK
361 AQLTRLNHIT NCNGAVLSVE ELLIGGEMIK AGTLTLEEVR RKFNNGEINF RS*
```

**AAVS1 ZFN SBS#30054 (CKKR):**

```
  1 MDYKDHDGDY KDHDIDYKDD DDKMAPKKKR KVGIHGVPAA MAERPFQCRI CMRNFSDRSN
 61 LSRHIRTHTG EKPFACDICG RKFALKQHLT RHTKIHTHPR APIPKPFQCR ICMRNFSTSG
121 NLTRHIRTHT GEKPFACDIC GRKFARRDWR RDHTKIHTGS QKPFQCRICM RNFSQSSHLT
181 RHIRTHTGEK PFACDICGRK FARLDNRTAH TKIHLRGSQL VKSELEEKKS ELRHKLKYVP
241 HEYIELIEIA RNSTQDRILE MKVMEFFMKV YGYRGKHLGG SRKPDGAIYT VGSPIDYGVI
301 VDTKAYSGGY NLPIGQADEM QRYVKENQTR NKHINPNEWW KVYPSSVTEF KFLFVSGHFK
361 GNYKAQLTRL NRKTNCNGAV LSVEELLIGG EMIKAGTLTL EEVRRKFNNG EINF*
```

**Supplementary Figure S1. Amino acid sequences of the *AAVS1* ZFN pair used in this study.**

**Supplementary Figure S2**

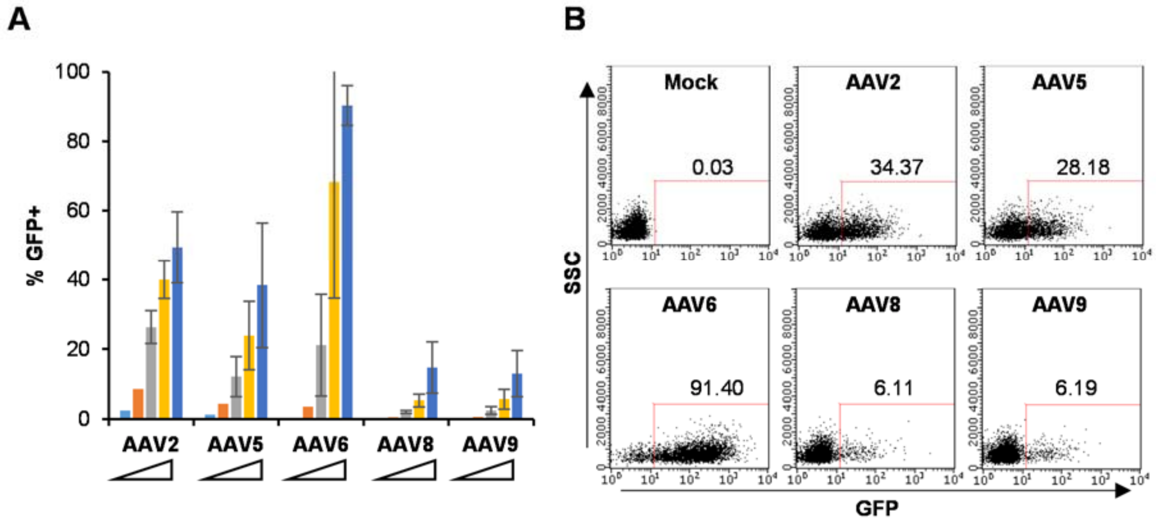


**Supplementary Figure S2. CD4⁺ T cells are efficiently transduced by AAV serotype 6.** CD4⁺ T cells were transduced with increasing doses of GFP-expressing AAV vectors of the indicated serotypes, and GFP expression was determined at 5 days post-transduction by flow cytometry. The vector doses used were $1 \times 10^4$, $3 \times 10^4$, $1 \times 10^5$, $3 \times 10^5$, and $1 \times 10^6$ vector genomes (vg)/cell. (**A**) Combined results of 3 independent experiments using different CD4⁺ T cell donors were shown as mean +/- SD. (**B**) Flow cytometric plots from a representative experiment at $3 \times 10^5$ vg/cell are shown.

**Supplementary Figure S3**

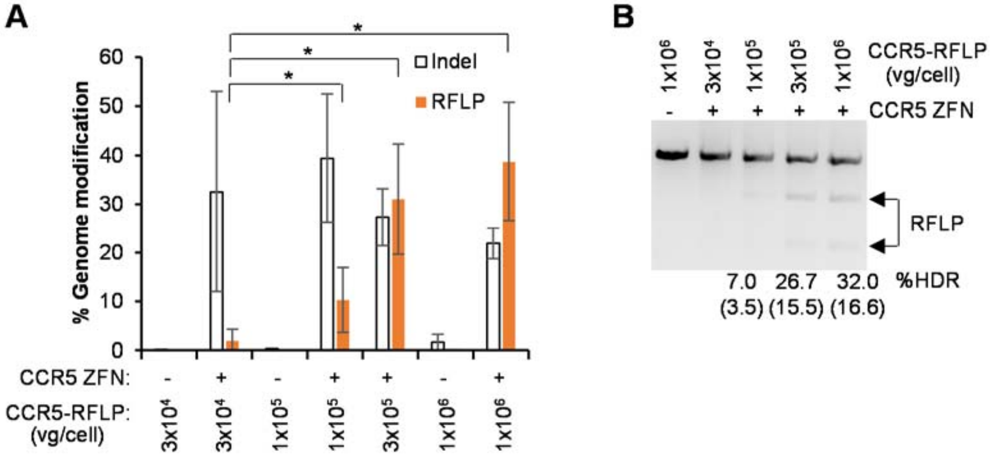


**Supplementary Figure S3. Combination of ZFN mRNA and AAV6 vectors promotes high levels of gene correction-like events at the *CCR5* locus in CD4⁺ T cells.** (**A**) CD4⁺ T cells were transduced with AAV6 vectors carrying the *CCR5*-RFLP donor at indicated doses for 16 hours, then electroporated with *CCR5* ZFN mRNA (60 µg/ml). Cells were analyzed 3-4 days post-electroporation by deep sequencing to measure the efficiency of genome modification (% indels and RFLP). Combined results of 6 experiments using 6 different CD4⁺ T cell donors are shown as mean +/- SD. * $p<0.05$, 2-tailed t-test to compare %RFLP HDR between conditions with different doses of AAV6 donor in the presence of ZFN mRNA treatment. (**B**) Dose-dependent insertion of XhoI site at *CCR5*, confirmed by RFLP analysis. Adjusted %RFLP HDR are shown below the lanes with visible RFLP bands based on deep sequencing results of the undigested top band due to incomplete digestion. The %RFLP before adjustment are shown in parentheses. One representative experiment is shown.

**Supplementary Figure S4**

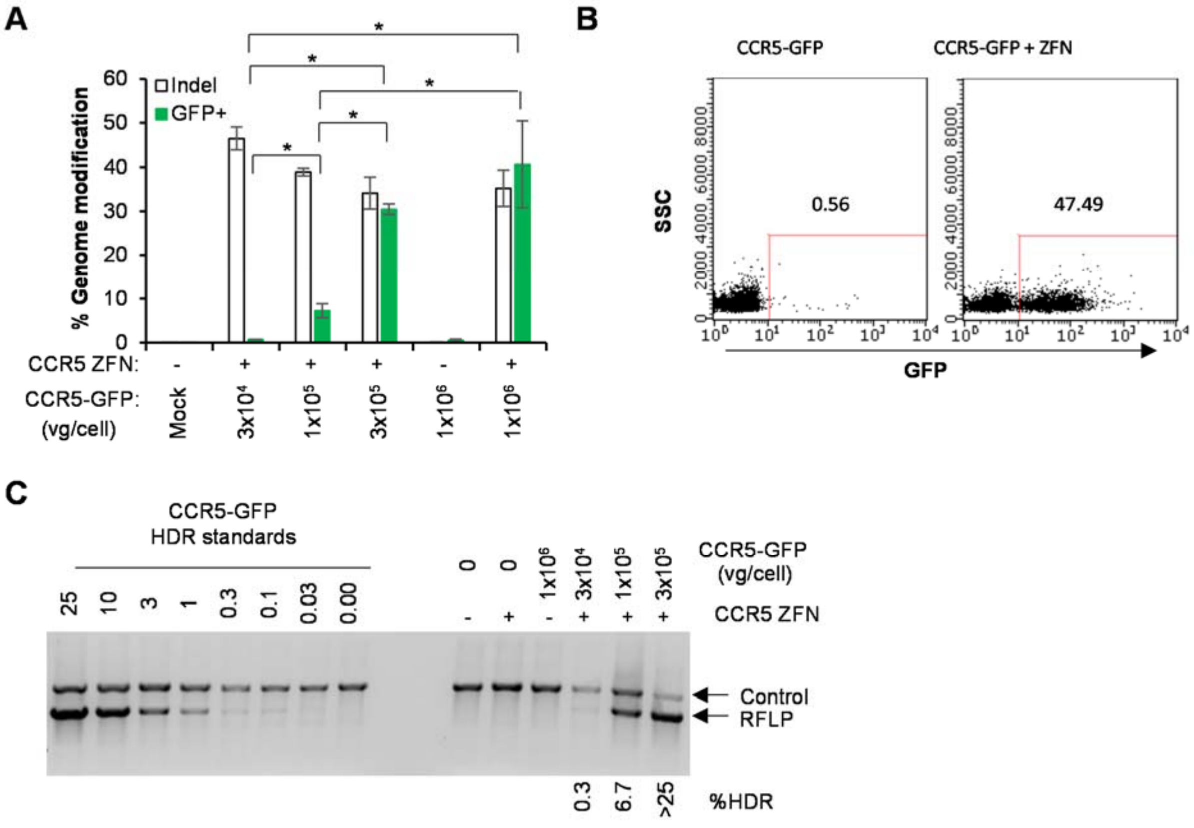


**Supplementary Figure S4. Combination of ZFN mRNA and AAV6 vectors promotes high levels of transgene addition at the *CCR5* locus in CD4$^+$ T cells.** (**A**) CD4$^+$ T cells were treated as described in **Supplementary Figure 2A**, but using *CCR5*-GFP donor vectors, with and without *CCR5* ZFN mRNA electroporation. Cells were collected at 7 days post-transduction and analyzed by flow cytometry for % GFP$^+$. Results are combined from two experiments using different CD4$^+$ T cell donors, and show mean +/- SD. * p<0.05, 2-tailed t-test to compare %GFP+ between conditions with different doses of AAV6 donor in the presence of ZFN mRNA treatment. (**B**) Flow cytometry plots from one representative experiment using 1 x $10^6$ vg/cell *CCR5*-GFP donor, at 7 days post-electroporation. (**C**) Confirmation of targeted integration of GFP expression cassette at the *CCR5* locus by semi-quantitative PCR. The % GFP HDR was estimated by comparison to standards.

**Supplementary Figure S5**

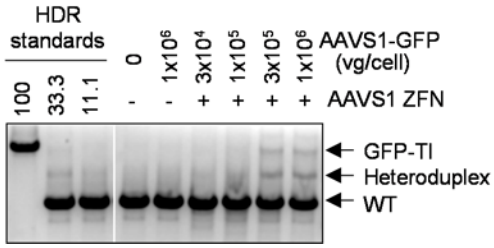


**Supplementary Figure S5: Confirmation of high levels of transgene addition at the *AAVS1* locus in CD8+ T cells.** CD8+ T cells were transduced with AAV6 vectors carrying the *AAVS1*-GFP donor at indicated doses (vg/cell) for 16 hours and/or electroporated with *AAVS1* ZFN mRNA. Cells were analyzed 14 days post-electroporation by an Out-Out PCR as described in Materials And Methods to detect targeted integration (TI) of GFP at the *AAVS1* locus by comparison to the HDR standards. The presence of GFP-TI is indicated by the top arrow.

**Supplementary Figure S6**

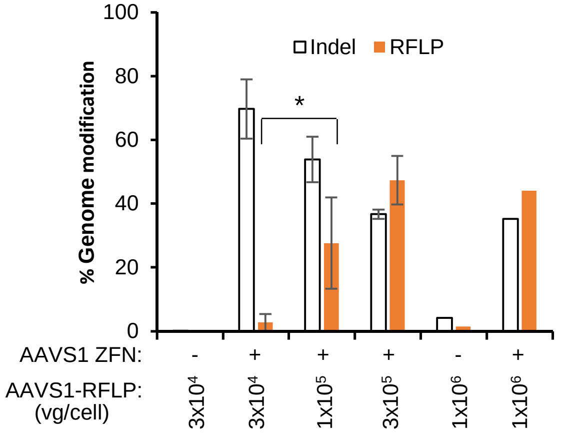


**Supplementary Figure S6: AAV6 vectors and ZFN mRNA promote high levels of gene editing at the *AAVS1* locus in $CD4^+$ T cells.** $CD4^+$ T cells were transduced with AAV6 vectors carrying the *AAVS1*-RFLP donor at indicated doses (vg/cell) for 16 hours and/or electroporated with *AAVS1* ZFN mRNA. Cells were analyzed 4 days post-electroporation by Illumina deep sequencing to measure the efficiency of genome modification (% indels and RFLP). Results are combined from 3 experiments using different $CD4^+$ T cell donors, and show mean +/- SD. * $p<0.05$, 2-tailed t-test to compare %RFLP HDR between conditions with different doses of AAV6 donor in the presence of ZFN mRNA treatment.

**Supplementary Figure S7**

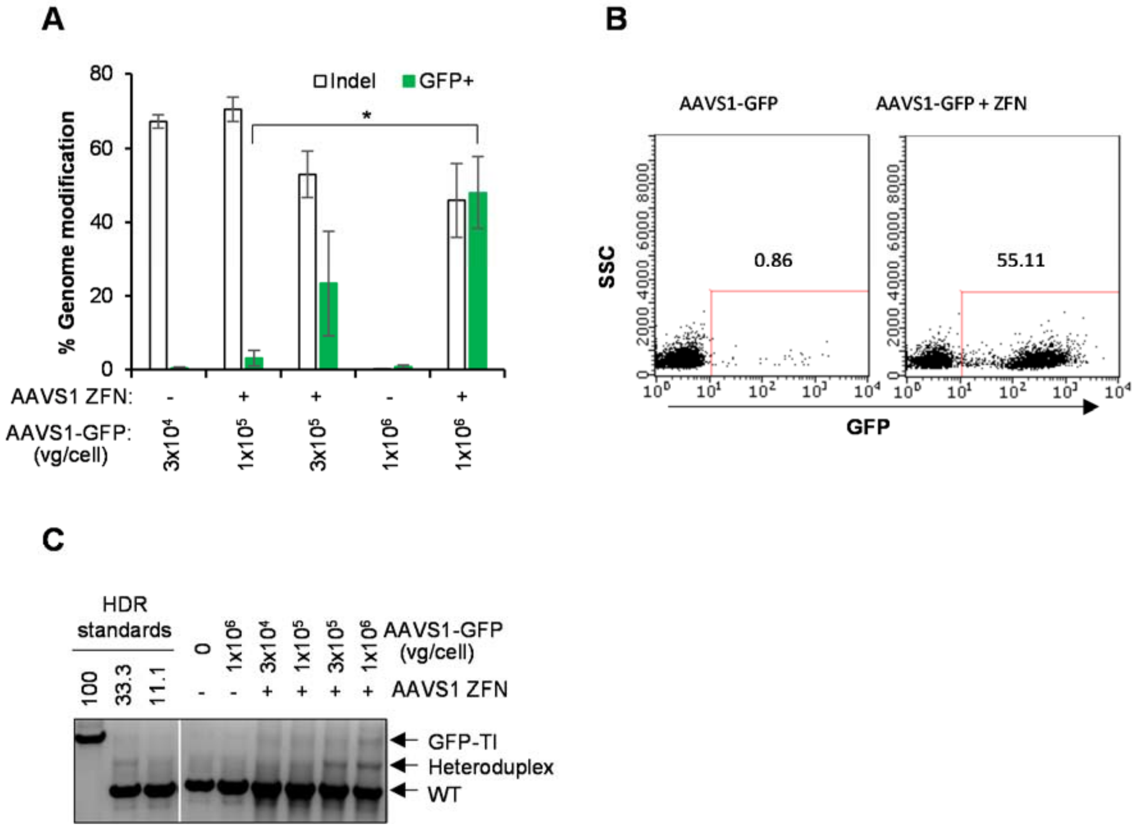


**Supplementary Figure S7: AAV6 vectors and ZFN mRNA promote high levels of transgene addition at the *AAVS1* locus in CD4$^+$ T cells.** (**A**) CD4$^+$ T cells were treated as described in **Supplementary Figure 5**, but using *AAVS1*-GFP donor. Cells were collected 7 days post-transduction and analyzed by flow cytometry for % GFP$^+$, and by deep sequencing to measure % indels. Results are combined from two experiments using different CD4$^+$ T cell donors, and show mean +/- SD. * p<0.05, 2-tailed t-test to compare %GFP+ between conditions with different doses of AAV6 donor in the presence of ZFN mRNA treatment. (**B**) Flow cytometry plots from a representative experiment using 1 x $10^6$ vg/cell *AAVS1*-GFP donor at 7 days post-electroporation. (**C**) Confirmation of targeted integration of GFP expression cassette at the *AAVS1* locus by an out-out PCR. The presence of GFP-TI is indicated by the top arrow.

**Supplementary Figure S8**

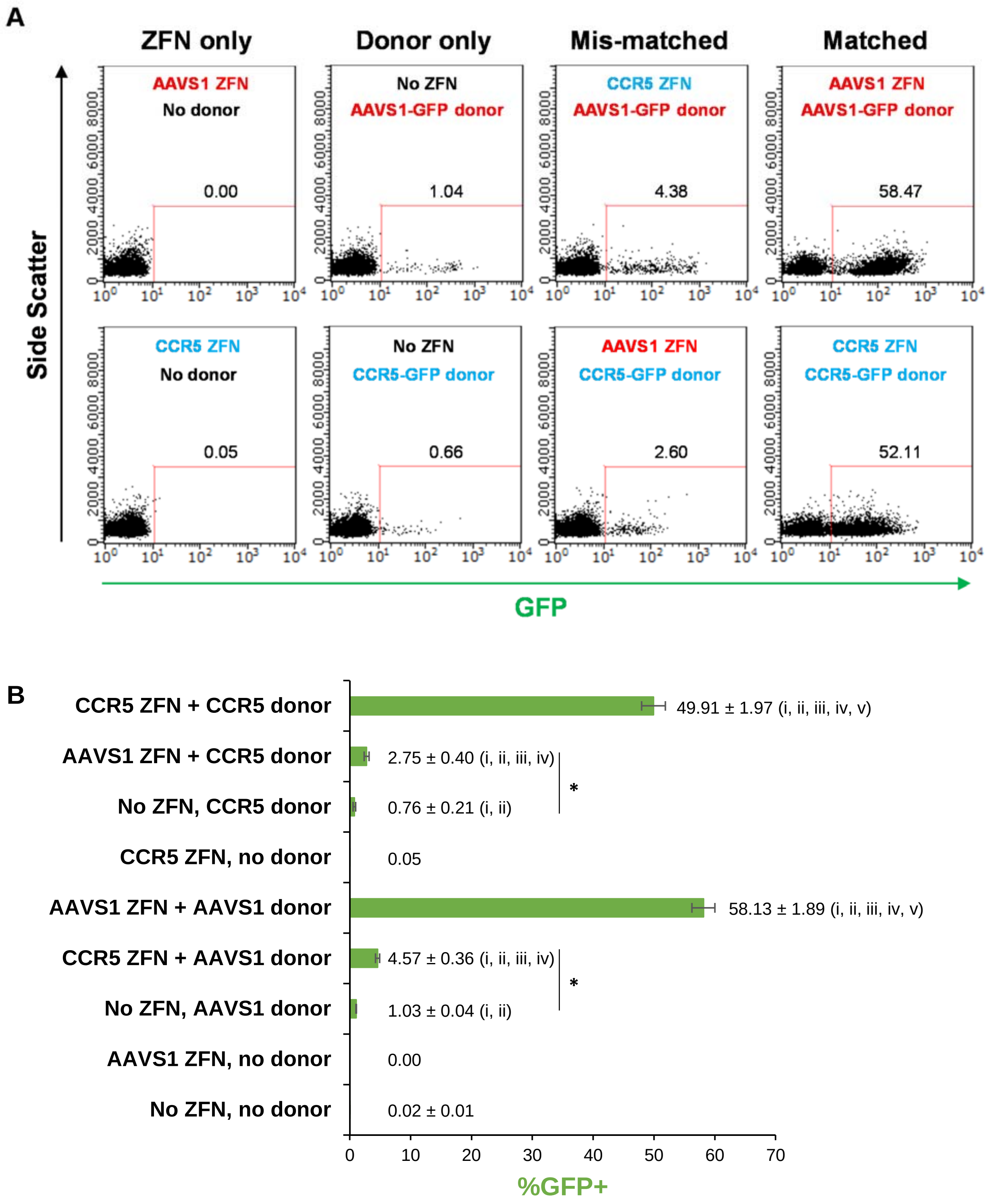


**Supplementary Figure S8. HDR is the predominant DNA repair pathway in genome editing mediated by delivery of ZFN as mRNA and donor as AAV6 vector in T cells.** Primary CD3$^+$ T cells were transduced

without or with AAV6 vectors carrying the AAVS1-GFP or CCR5-GFP donor at 1 X $10^6$ vg/cell for 16 hours and/or electroporated with 20 ug/ml AAVS1 or 40ug/ml CCR5 ZFN mRNA. Cells were expanded and analyzed 9 days post-electroporation by flow cytometry. (**A**) One set of representative FACS plots are shown. (**B**) Results (mean ± SD) are from triplicated samples except the “AAVS1 ZFN, no donor” and “CCR5 ZFN, no donor” treatment conditions. * $P < 0.05$, one-way ANOVA. The %GFP+ detected in samples treated with “No ZFN, AAVS1-GFP donor” (1.03 ± 0.04%) and “No ZFN, CCR5-GFP donor” (0.76 ± 0.21%) represent GFP expression from episomal DNA (i) and random integration (ii) of AAV genome. The difference (3.54%) between “CCR5 ZFN + AAVS1-GFP donor” (4.57 ± 0.36%) and “No ZFN, AAVS1-GFP donor” (1.03 ± 0.04%) represent end capture (integration of AAV genome at a double-strand break site in cellular genome) mediated by CCR5 ZFN cleavage (iii & iv). The majority of the 3.54% are presumably located at the CCR5 on-target site (iii). A small portion of the 3.54% are presumably located at some off-target sites (iv). The difference (55.38%) between “AAVS1 ZFN + AAVS1-GFP donor” (58.13 ± 1.89%) and “AAVS1 ZFN + CCR5-GFP donor” (2.75 ± 0.40%) represent HDR (v). The difference (1.99%) between “AAVS1 ZFN + CCR5-GFP donor” (2.75 ± 0.40%) and “No ZFN, CCR5-GFP donor” (0.76 ± 0.21%) represent end capture mediated by AAVS1 ZFN cleavage (iii & iv). The majority of the 1.99% are presumably located at the AAVS1 on-target site (iii). A small portion of the 1.99% are presumably located at some off-target sites (iv). The difference (45.34%) between “CCR5 ZFN + CCR5-GFP donor” (49.91 ± 1.97%) and “CCR5 ZFN + AAVS1-GFP donor” (4.57 ± 0.36%) represent HDR (v).

**Supplementary Figure S9**

A

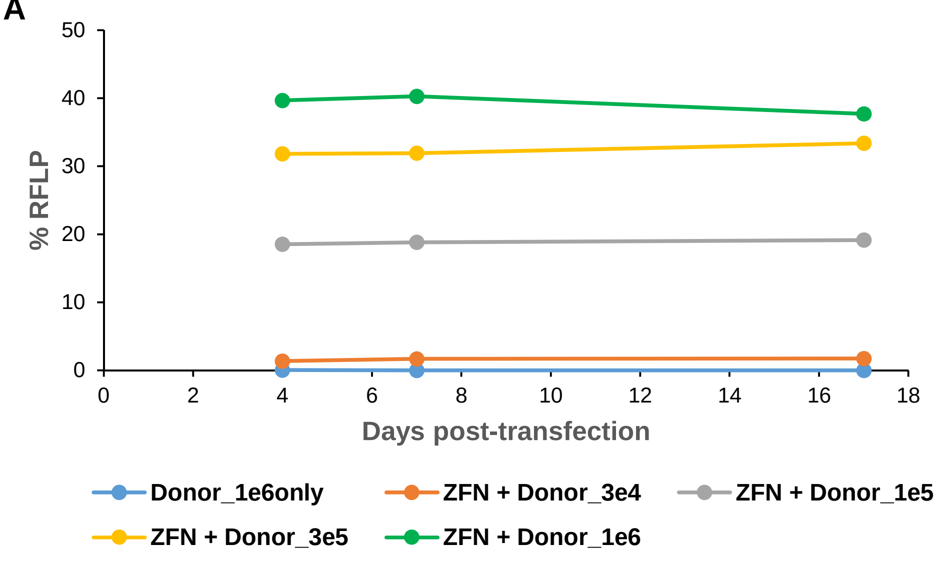


B

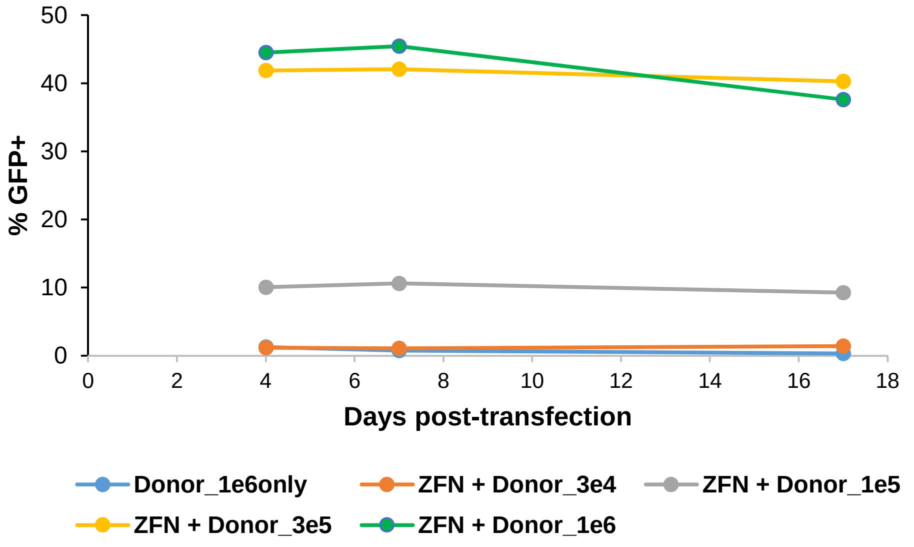


**Supplementary Figure S9. Stability of AAV6 vectors and ZFN mRNA mediated genome editing.** (**A**) CD8$^{+}$ T cells were transduced with AAV6 vectors carrying the *CCR5*-RFLP donor at indicated doses (vg/cell) for 16 hours, then electroporated with *CCR5* ZFN mRNA (60 µg/ml). Cells were analyzed at indicated time points post-electroporation by deep sequencing to measure the efficiency of genome modification (% RFLP). (**B**) CD4$^{+}$ T cells were treated as described above, but using *CCR5*-GFP donor. Cells were collected at indicated time points and analyzed by flow cytometry for % GFP$^{+}$.

**Supplementary Figure S10**

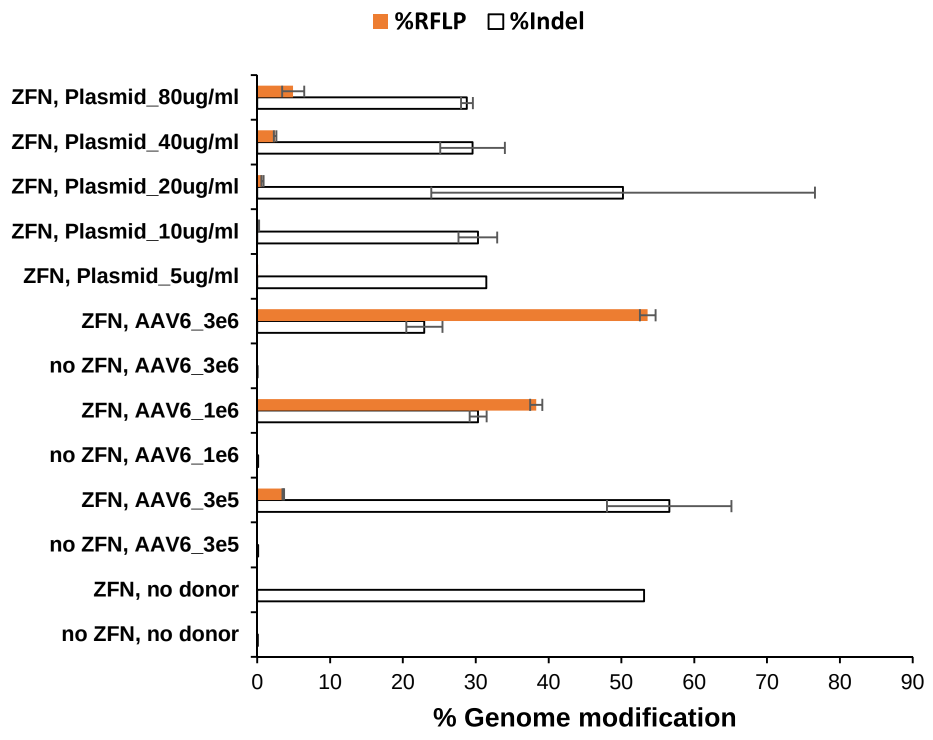


**Supplementary Figure S10: Higher levels of HDR supported by AAV6 than plasmid donor.** $CD3^+$ T cells were transduced with AAV6 vectors carrying the *CCR5*-RFLP donor at doses of 3 – 3.3 folds serial dilutions for 16 hours and then electroporated with *CCR5* ZFN mRNA, or cells were electroporated with indicated amount of a *CCR5*-RFLP plasmid DNA donor together with *CCR5* ZFN mRNA. Cells were analyzed at 5 days post-electroporation by Illumina deep sequencing to measure the efficiency of genome modification (% indel and %RFLP). Results shown are mean +/- SD of duplicated samples.

**Supplementary Figure S11**

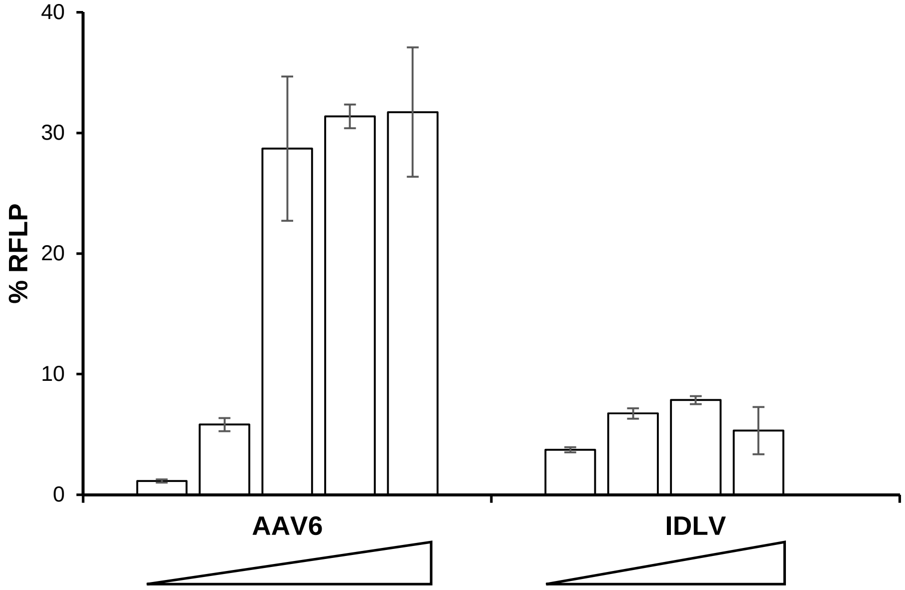


**Supplementary Figure S11: Higher levels of HDR supported by AAV6 than IDLV donor.** CD4⁺ T cells were transduced with AAV6 or integrase-deficient lentivirus (IDLV) vectors carrying the *CCR5*-RFLP donor at doses of 3 – 3.3 folds serial dilutions for 16 hours and then electroporated with *CCR5* ZFN mRNA. Cells were analyzed at 4 days post-electroporation by Illumina deep sequencing to measure the efficiency of genome modification (% RFLP). Results shown are mean +/- SD of duplicated samples.

**Supplementary Figure S12**

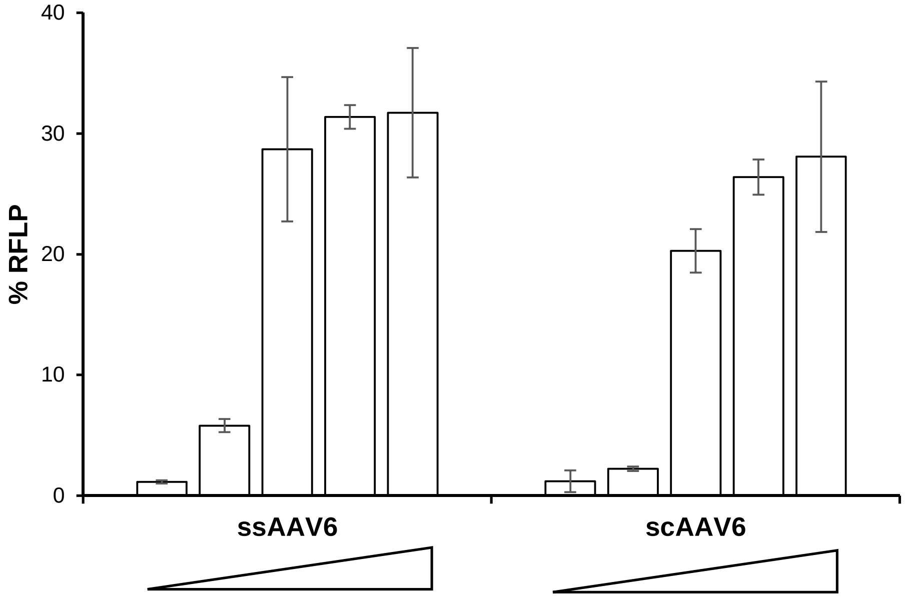


**Supplementary Figure S12. Comparable levels of HDR stimulated by single-stranded (ss) or self-complementary (sc) AAV6 donor.** CD4+ T cells were transduced with ssAAV6 or scAAV6 vectors carrying the *CCR5*-RFLP donor at doses of 3 – 3.3 folds serial dilutions for 16 hours and then electroporated with *CCR5* ZFN mRNA. Cells were analyzed at 4 days post-electroporation by Illumina deep sequencing to measure the efficiency of genome modification (% RFLP). Results shown are mean +/- SD of duplicated samples.

**Supplementary Figure S13**

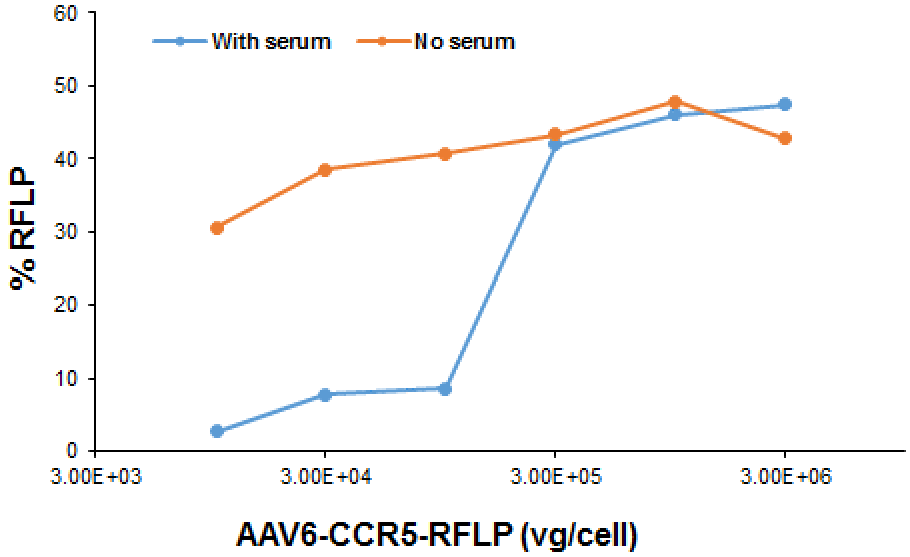


**Supplementary Figure S13. AAV6 donor transduction under serum-free conditions leads to a marked improvement in HDR-driven genome editing at lower doses of vector.** CD3$^+$ T cells were transduced with AAV6 *CCR5*-RFLP donor vector at doses ranging from 1 x $10^4$ to 3 x $10^6$ vg/cell, using vector serially diluted by 3 – 3.3 fold, for 4 hours under regular serum-containing (with serum) or serum-free (no serum) media and then electroporated with *CCR5* ZFN mRNA (60ug/ml). Cells were maintained and expanded for 5 days in regular serum-containing media post-electroporation, then collected and analyzed by Illumina deep sequencing to measure the efficiency of genome modification (% RFLP).

## Supplementary Table

### **Supplementary Table S1. Primers used for *CCR5* and *AAVS1* analysis.**

| Target | PCR | Forward Primer | Reverse Primer |
|---|---|---|---|
| **Out-Out PCR primers** | | | |
| *CCR5* | out-out1 | CTGTGCTTCAAGGTCCTTGTCTGC | CTCTGTCTCCTTCTACAGCCAAGC |
| | out-out2 | GCACCATGCTTGACCCAGTTTCTTA | CAAGTCTCTCGCCTGGTTCTAAGTCA |
| *AAVS1* | out-out1 | CGGAACTCTGCCCTCTAACG | CTGGGATACCCCGAAGAGTG |
| | out-out2 | CGTCTCTCTCCTGAGTCCG | CACAGTTGGAGGAGAATCCACC |
| **MiSeq adaptor PCR primers*** | | | |
| *CCR5* | adaptor | NGCCAGGTTGAGCAGGTAGATG | NGCTCTACTCACTGGTGTTCATCTTT |
| *AAVS1* | adaptor | CTCTGGTTCTGGGTACTTTTATCTG | ACTGACATCTACCTGCTCAACC |
| **In-Out PCR primers** | | | |
| *CCR5* | in-out | GAGGATTGGGAAGACAATAGCAG | CCAGCAATAGATGATCCAACTCAAATTCC |
| | control | GATTTGCACAGCTCATCTGGC | CCATCTTGTTCCACCCTGTGC |

* Only the target-specific portion of the MiSeq adaptor PCR primers is listed.
Forward primer sequence is preceded by 5'- ACACGACGCTCTTCCGATCTNNNN.
Reverse primer sequence is preceded by 5'- GACGTGTGCTCTTCCGATCT.

**Supplementary Table S2. Alignment of the top 20 sequences detected in a sample treated with CCR5-RFLP donor and CCR5 ZFN**

| Seq# | % | Type | Sequence alignment* |
|---|---|---|---|
| 1 | 41.15 | Wild-type | GTCATGCTCTTCAGCCTTTTGCAGTTTATCAGGATGAGGATGACCAGCATGTTGCCCAC |
| 2 | 42.80 | HDR | GTCATGCTCTTCAGCCTTTTGCAGTTTGTCTCGAGGTGATGAGGATGACCAGCATGTTGCCCAC |
| 3 | 2.86 | +5bp | GTCATGCTCTTCAGCCTTTTGCAGTTTATCAGATCAGGATGAGGATGACCAGCATGTTGCCCAC |
| 4 | 1.05 | +1bp | GTCATGCTCTTCAGCCTTTTGCAGTTTATCAGGGATGAGGATGACCAGCATGTTGCCCAC |
| 5 | 0.51 | -14bp | GTCATGCCCTTCAGCCTTTTGCAG--------------GATGACCAGCATGTTGCCCAC |
| 6 | 0.44 | -5bp | GTCATGCTCTTCAGCCTTTTGCAGTTT-----GATGAGGATGACCAGCATGTTGCCCAC |
| 7 | 0.42 | -1bp | GTCATGCTCTTCAGCCTTTTGCAGTTTATCAG-ATGAGGATGACCAGCATGTTGCCCAC |
| 8 | 0.41 | -23bp | GTCATGCTCTTCAGCCTTTTGCAG-----------------------CATGTTGCCCAC |
| 9 | 0.38 | +3bp | GTCATGCTCTTCAGCCTTTTGCAGTTTATCATCAGGATGAGGATGACCAGCATGTTGCCCAC |
| 10 | 0.37 | -3bp | GTCATGCTCTTCAGCCTTTTGCAGTTTA---GGATGAGGATGACCAGCATGTTGCCCAC |
| 11 | 0.35 | -9bp | GTCATGCTCTTCAGCCTTTTGCAGTT---------GAGGATGACCAGCATGTTGCCCAC |
| 12 | 0.33 | +4bp | GTCATGCTCTTCAGCCTTTTGCAGTTTATTAATCAGGATGAGGATGACCAGCATGTTGCCCAC |
| 13 | 0.26 | -2bp | GTCATGCTCTTCAGCCTTTTGCAGTTT--CAGGATGAGGATGACCAGCATGTTGCCCAC |
| 14 | 0.25 | +2bp | GTCATGCTCTTCAGCCTTTTGCAGTTTATATCAGGATGAGGATGACCAGCATGTTGCCCAC |
| 15 | 0.24 | -6bp | GTCATGCTCTTCAGCCTTTTGCAGTTTATCAGGATGA------CCAGCATGTTGCCCAC |
| 16 | 0.21 | -8bp | GTCATGCTCTTCAGCCTTTTGCAG--------GATGAGGATGACCAGCATGTCGCCCAC |
| 17 | 0.20 | -9bp | GTCATGCTCTTCAGCCTTTTGCAG---------ATGAGGATGACCAGCATGTTGCCCAC |
| 18 | 0.20 | -46bp | G----------------------------------------------CATGTGGCCCAC |
| 19 | 0.18 | -8bp | GTCATGCTCTTCAGCCTTTTGCAGTTT--------GAGGATGACCAGCATGTTGCCCAC |
| 20 | 0.18 | -15bp | GTCATGCTCTTCAGCCTTTTG---------------AGGATGACCAGCATGTTGCCCAC |

Supplementary Table S2. $CD8^+$ T cells were transduced with 1 X $10^6$ vg/cell AAV6 vectors carrying the CCR5-RFLP donor for 16 hours, then electroporated with CCR5 ZFN mRNA (60 µg/ml). Cells were analyzed 7 days post-electroporation by deep sequencing to measure the efficiency of genome modification (% indels and RFLP HDR). The top 20 sequences from a treated sample were aligned against the wild-type CCR5 sequence. Only the most important portion of the sequences is shown. In sequence alignment, CCR5 ZFN binding sites are underlined. Deleted bases are indicated by "-". Inserted bases are indicated in red color. *One representative sequence for each type of alignment is shown.

**Supplementary Table S3. Alignment of the top 20 sequences detected in a sample treated with AAVS1-RFLP donor and AAVS1 ZFN**

| Seq# | % | Type | Sequence alignment* |
|---|---|---|---|
| 1 | 15.06 | Wild-type | TTTATCTGTCCCCTCCACCCCACAGTGGGGCCACTAGGGACAGGATTGGTGACAGAAAAGCCC |
| 2 | 63.69 | HDR | TTTATCTGTCCCCTCCACCCCACAGTGGGGCAAGCTTTACTAGGGACAGGATTGGTGACAGAAAAGCCC |
| 3 | 1.81 | -10bp | TTTATCTGTCCCCTCCACCCCACAGTGGG----------ACAGGATTGGTGACAGAAAAGCCC |
| 4 | 1.77 | +4bp | TTTATCTGTCCCCTCCACCCCACAGTGGGGCCAGCCACTAGGGACAGGATTGGTGACAGAAAAGCCC |
| 5 | 0.94 | -21bp | TTTATCTGTCCCCTCCACCCCACAGTGG---------------------TGACAGAAAAGCCC |
| 6 | 0.76 | -1bp | TTTATCTGTCCCCTCCACCCCACAGTGGG-CCACTAGGGACAGGATTGGTGACAGAAAAGCCC |
| 7 | 0.64 | -3bp | TTTATCTGTCCCCTCCACCCCACAGTGGG---ACTAGGGACAGGATTGGTGACAGAAAAGCCC |
| 8 | 0.64 | -18bp | TTTATCTGTCCCCTCCACCCCACAG------------------GATTGGTGACAGAAAAGCCC |
| 9 | 0.59 | -4bp | TTTATCTGTCCCCTCCACCCCACAGTGGC----CTAGGGACAGGATTGGTGACAGAAAAGCCC |
| 10 | 0.58 | -11bp | TTTATCTGTCCCCTCCACCCCACAGTGG-----------ACGGGATTGGTGACAGAAAAGCCC |
| 11 | 0.49 | -24bp | TTTATCTGTCCCCTCCACCCCACAGTG------------------------ACAGAAAAGCCC |
| 12 | 0.43 | -5bp | TTTATCTGTCCCCTCCACCCCACAGTG-----ACTAGGGACAGGATTGGTGACAGAAAAGCCC |
| 13 | 0.41 | -13bp | TTTATCTGTCCCCTCCACCCCACAG-------------GACAGGATAGGTGACAGAAAAGCCC |
| 14 | 0.39 | -2bp | TTTATCTGTCCCCTCCACCCCACAGTGGGGCCA--AGGGACAGGATTGGTGACAGAAAAGCCC |
| 15 | 0.38 | -3bp | TTTATCTGTCCCCTCCACCCCACAGTGGGTTAG---GGGACAGGATTGGTGACAGAAAAGCCC |
| 16 | 0.38 | -7bp | TTTATCTGTCCCCTCCACCCCACAGTGGAGCCAC-------AGGATTGGTGACAGAAAAGCCC |
| 17 | 0.34 | -11bp | TTTATCTGTCCCCTCCACCATCC-----------TAGGGACAGGATTGGTGACAGAAAAGCCC |
| 18 | 0.32 | -2bp | TTTATCTGTCCCCTCCACCCCACAGTGG--CCACTAGGGACAGGATTGGTGACAGAAAAGCCC |
| 19 | 0.32 | -9bp | TTTATCTGTCCCCTCCACCCCACAGTGGGG---------ACAGGATTGGTGACAGAAAAGCCC |
| 20 | 0.30 | -33bp | TTTATCTGTCCCCTCCACCCC---------------------------------GAAAAGCCC |

Supplementary Table S3. $CD8^{+}$ T cells were transduced with $3 \times 10^{5}$ vg/cell AAV6 vectors carrying the AAVS1-RFLP donor for 16 hours, then electroporated with AAVS1 ZFN mRNA (40 µg/ml). Cells were analyzed 7 days post-electroporation by deep sequencing to measure the efficiency of genome modification (% indels and RFLP HDR). The top 20 sequences from a treated sample were aligned against the wild-type AAVS1 sequence. Only the most important portion of the sequences is shown. In sequence alignment, AAVS1 ZFN binding sites are underlined. Deleted bases are indicated by "-". Inserted bases are indicated in red color. *One representative sequence for each type of alignment is shown.
